# Insights into the Molecular Mechanisms Regulating Cell Behavior in Response to Magnetic Materials and Magnetic Stimulation in Stem Cell (Neurogenic) Differentiation

**DOI:** 10.3390/ijms24032028

**Published:** 2023-01-19

**Authors:** Alexandra-Elena Mocanu-Dobranici, Marieta Costache, Sorina Dinescu

**Affiliations:** 1 Department of Biochemistry and Molecular Biology, University of Bucharest, 050095 Bucharest, Romania; 2 Research Institute of the University of Bucharest (ICUB), 050063 Bucharest, Romania

**Keywords:** magnetic nanoparticles, magnetic stimulation, tissue engineering, stem cell differentiation, cytoskeleton, epigenetic changes

## Abstract

Magnetic materials and magnetic stimulation have gained increasing attention in tissue engineering (TE), particularly for bone and nervous tissue reconstruction. Magnetism is utilized to modulate the cell response to environmental factors and lineage specifications, which involve complex mechanisms of action. Magnetic fields and nanoparticles (MNPs) may trigger focal adhesion changes, which are further translated into the reorganization of the cytoskeleton architecture and have an impact on nuclear morphology and positioning through the activation of mechanotransduction pathways. Mechanical stress induced by magnetic stimuli translates into an elongation of cytoskeleton fibers, the activation of linker in the nucleoskeleton and cytoskeleton (LINC) complex, and nuclear envelope deformation, and finally leads to the mechanical regulation of chromatin conformational changes. As such, the internalization of MNPs with further magnetic stimulation promotes the evolution of stem cells and neurogenic differentiation, triggering significant changes in global gene expression that are mediated by histone deacetylases (e.g., HDAC 5/11), and the upregulation of noncoding RNAs (e.g., miR-106b~25). Additionally, exposure to a magnetic environment had a positive influence on neurodifferentiation through the modulation of calcium channels’ activity and cyclic AMP response element-binding protein (CREB) phosphorylation. This review presents an updated and integrated perspective on the molecular mechanisms that govern the cellular response to magnetic cues, with a special focus on neurogenic differentiation and the possible utility of nervous TE, as well as the limitations of using magnetism for these applications.

## 1. Introduction

Recently, special attention has been paid to the potential use of magnetic materials and magnetic stimulation for tissue reconstruction [1]. The use of magnetic nanoparticles (MNPs) in the development of biomaterials for tissue engineering (TE) applications is justified by their good biocompatibility [2] and tunable magnetic properties [3]. Broadly, MNPs can be obtained from different types of metal alloys based on Fe, Ni, Co, and Ti; from iron oxides; or from ferrite. However, even though such diverse choices are available, iron oxides, such as magnetite (Fe_4_O_3_) or maghemite (α-Fe_2_O_3_), are the preferred choice due to their superior biocompatibility and low cytotoxic effects. Other advantages of MNPs are the presence of surface functional groups, which allow for the grafting of bioactive compounds, and have the ability to direct these compounds with the aid of magnetic forces and tailorable physical properties [4,5]. Additionally, MNPs based on iron oxides lose magnetization after the removal of the applied magnetic field, making them suitable for biomedical applications [6]. Thanks to their superparamagnetic properties, MNPs can be manipulated with the use of an external magnetic field, allowing them to be accumulated in specific locations in the body. This feature makes them suitable for various biomedical applications such as imaging, biosensors, cancer therapy, drug and gene delivery, and tissue engineering by ensuring local and controlled administration [4,5,7]. In order to obtain scaffolds with magneto-responsive properties, MNPs are usually embedded into their matrices. The incorporation of MNPs also helps with improving mechanical properties, especially in the case of scaffolds with a lower rigidity, such as hydrogels [1,8]. Additionally, magnetic fields have proved to be useful in controlling cellular adhesion, stimulating stem cell proliferation and differentiation [1,9], and impairing cancer cells’ migration [10]. Magnetic stimulation can be combined with the use of magnetic materials in order to enhance the biological response of the cells and to direct their growth and orientation in order to mimic structures with highly intricate architectures [1]. Another focus for this type of approaches is the possibility to apply them to nervous TE. The complexity of the nervous system and its poor regenerative capacity are the main reasons why, in this case, tissue reconstruction represents a huge challenge and requires the development of new strategies with therapeutic potential. MNPs, along with magnetic scaffolds and magnetic actuation, have recently emerged as promising players in nervous TE since beneficial effects were reported regarding nervous cell response and neuronal differentiation [11,12].

Even though an upsurge in the use of magnetic materials and actuation for TE purposes can be observed, there are still unknown questions regarding the in-depth mechanisms that govern their biological effects. Therefore, the aim of this paper is to review how these types of cues influence cell behavior at a molecular level, how they impact the cytoskeleton network and nuclear morphology, and how they consequently correlate with epigenetic reprogramming and signaling pathways during stem cell differentiation. Furthermore, in the last section we discuss the current knowledge and applications concerning their use for nervous tissue regeneration.

## 2. Interactions with Magnetic Materials and Magnetic Fields Can Impact Cellular Morphology with Implications for the Focal Adhesion–Cytoskeleton–Nuclear Membrane Axis

**Figure 1:**
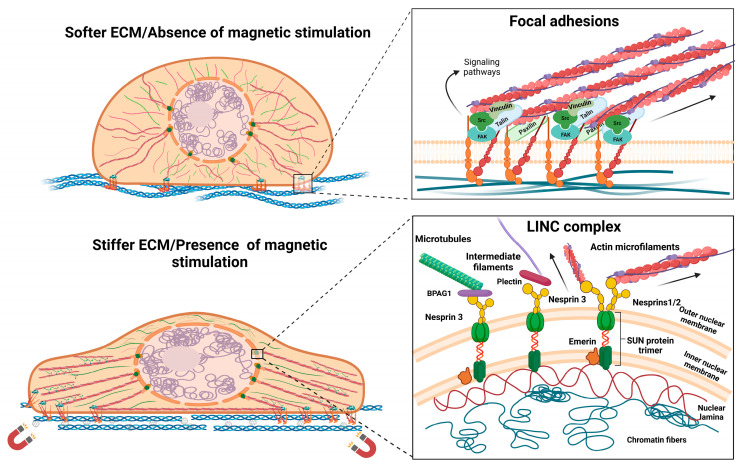
Morphological changes after interaction with magnetic materials and magnetic stimulation, representing the focal adhesion–cytoskeleton–nuclear membrane axis. Abbreviations: bullous pemphigoid antigen—BPAG1; extracellular matrix—ECM; focal adhesion kinase—FAK; linker of nucleoskeleton and cytoskeleton—LINC; Sad/UNC84—SUN domain proteins. Image was created with BioRender.com.

Given the high variety of scaffolds used tissue reconstruction, one particular topic of interest in regard to TE approaches is how cells respond to different types of substrates and stimuli, as they usually respond through focal adhesion (FA) redistribution and assembly, and by changing their morphology [13,14]. Characteristics such as the stiffness of the substrate, its micro- and nanoarchitecture, its conductivity, and other biophysical properties were shown to regulate cells’ interaction with the substrate and the biological response. These features have an impact on FA, which is how protein complexes mediate cells’ contact with their microenvironment [13,15,16,17]. FAs are integrin-based dynamic structures of the cells that can be assembled and disassembled in response to environmental conditions or during processes such as cellular migration. Besides integrins that directly interact with the substrate, FAs contain structural proteins (among them, paxillin, talin, and vinculin), as well as functional proteins such as focal adhesion kinase (FAK), that activate signaling pathways [18]. Mechanical stimuli and extracellular signal-regulated kinase (ERK)-signaling activation through FAs seem to have important roles in the differentiation of stem cells toward osteogenic [19] and neurogenic [16] lineages. Additionally, the complex network of cytoskeletal proteins, especially actin microfilaments, is strongly connected to FA complexes by structural proteins, as represented in Figure 1. Therefore, mechanical stimuli at FA level are usually propagated through cytoskeleton and signaling pathways activated by FA can influence its distribution. However, cytoskeleton changes can equally impact FA complexes [15,16,17,18]. Furthermore, changes occurring in the cytoskeleton architecture and dynamics also play a significant role during the differentiation process, especially when they are associated with significant modifications to the cellular morphology [20,21,22].

Regarding the influence of magnetic cues on cellular behavior, there are numerous studies focusing on the reorganization of the cytoskeleton network and the effects on the focal adhesion complex or gap junctions [23,24,25]. It has been shown that a magnetic field and high concentrations of MNPs can lead to a decrease in proliferation and negatively affect FA and cytoskeleton components [26,27]. From this perspective, magnetic stimulation has emerged as a potential therapy that can affect cancer cells by interfering with processes such as cell migration and motility [28]. However, when used in a controlled manner and under optimized conditions, it was proven to have a positive influence on cytoskeleton distribution and complexes that mediate the cellular interaction with its environment, showing great potential in tissue regeneration applications [29,30]. Recent investigations reported short-term cell stiffening after the internalization of MNPs, along with actin reorganization and a transient increase in actin bundles, which is dependent on the concentration and exposure time. Such temporary changes might be explained in the context of endocytosis, as cells tend to return to the original state after complete internalization [31]. Mesenchymal stem cells (MSCs) grown on scaffolds embedded with MNPs presented an increased expression of integrins and signaling molecules downstream of the FAK/ERK pathway, which was accompanied by an overall improved adherence compared to that of simple scaffolds. This behavior might be explained by the enhanced biophysical properties of the scaffolds, which can be attributed to the incorporation of MNPs and to the magnetic field stimulation [32,33]. Studies on cellular morphology report that magnetic actuation enhances the anisotropy of plasma membranes, focal adhesions, and cytoskeletal structures [31,34]. The influence of magnetic cues on cellular morphology are represented in Figure 1. For dental pulp stem cells, exposure to a static magnetic field induced the alignment of fatty acid chains and, thus, increased plasma membrane rigidity, but these effects were reversed after the stimulation was ended [34]. Stem cells tend to adopt an elongated morphology as they are exposed to a magnetic field, since the alignment of filamentous actin increases [35]. Moreover, even though phenotypic changes cannot be observed, cytoskeleton reorganization is still present. Besides augmenting the anisotropy of cytoskeletal protein distribution, the presences of a magnetic field led to the thickening of actin filaments and stimulated polymerization [34]. Other approaches to modulating the cellular response through mechanical stimulation involved MNPs that were functionalized with specific sequences or peptides that target focal adhesions and surface receptors. After establishing interactions with these cellular compartments, MNPs were directed by using the magnetic field in order to exercise mechanical stress at the cell membrane level and induce morphological changes or activate signaling pathways [36,37]. The arginine-glycine-aspartate (RGD) motif found in the ECM functions as a binding site for integrins and is often used in such approaches to functionalize MNPs in order to mediate cell–nanoparticle interactions and control cellular behavior and stem cell differentiation [37,38].

Interestingly, magnetic stimulation was reported to modulate other surface proteins’ activity and the signaling pathways associated with them, such as the epidermal growth factor receptor (EGFR), with implications in stem cell differentiation [29] and changes occurring in actin [39] and microtubule [40] organization. Moreover, MNPs were shown to also influence cellular intercommunication. For example, Han et al. (2015) discovered that MNPs internalized by cardiomyoblasts lead to the upregulation of connexin 43 (Cx43), a gap junction protein that mediates contacts and communications between cells. In this case, Cx43 determined stronger interactions between cardiomyoblasts and MSCs, with the latter being more prone to adopt a cardiac phenotype and to express a paracrine profile with regenerative potential [23]. On the other hand, MSCs exposed to a static magnetic field seemed to respond by increasing the number of cellular projections between adjacent cells, such as lamellipodia and filopodia [41].

## 3. Influence on Nuclear Morphology and Localization

Besides being connected with integrins and FA complexes, the cytoskeletal proteins are anchored to the nuclear membrane via linker of the nucleoskeleton and cytoskeleton (LINC) complex that is located at the nuclear membrane level. LINC comprises two types of proteins: nesprins, which are spectrin repeat proteins, and Sad/UNC84 (SUN) domain proteins. Regarding their interaction with other cellular compartments, nesprins are localized at the outer nuclear membrane (ONM) level, where they mediate contact with cytoskeletal proteins. Nesprin-1 and -2 bind filamentous actin, whereas nesprin-3 binds with plectin which further binds with intermediate filaments, or it can interact with microtubules via the bullous pemphigoid antigen (BPAG1). There is also nesprin-4, which is known to be a kinesin-1-binding protein. Nesprins have a KASH (Klarsicht, ANC-1, Syne Homology) domain that allows interaction with SUN proteins, with inner nuclear membrane (INM) localization. SUN proteins, in turn, are directly connected to the lamins, which are a type of intermediate filament that offers structural stability to the nucleus [42,43,44]. Furthermore, emerin is another INM-localized protein in mammals that interacts with SUN trimers and participates in chromatin tethering. It has been demonstrated that in isolated nuclei, the phosphorylation of emerin by Src kinase occurs in response to mechanical stress through nesprin-1 and it is required for the recruitment of lamin A/C to the LINC complex [45]. Given the anchorage of the nucleus to the cytoskeletal proteins, this compartment has a high susceptibility to mechanical forces propagated through the cytoskeleton network, and components of the LINC complex impact focal adhesions and cytoskeleton organization [46,47]. The nucleus has been intensely studied as a mechanosensing organelle, as it responds to environmental cues by regulating its stiffness, morphology, or positioning [41,42,45,46,48,49]. Moreover, stem cell differentiation is accompanied by changes in nuclear plasticity. It seems that embryonic stem cells possess nuclei more susceptible to mechanical deformation and their rigidity gradually increases as they undergo neuroectodermal differentiation. On the other hand, with respect to nuclear stiffness, adult stem cells are defined by an intermediate state that is between pluripotent and terminal differentiated cells [48]. Studies have demonstrated that LINC components impact stem cells’ proliferative ability and differentiation when they regulate specification [50]. For example, during MSC differentiation into cardiomyocytes, nesprin-1 is overexpressed [50], whereas nesprin-2 is an important regulator during mechanical strain that induces the differentiation of fibroblasts into myofibroblasts [51]. Nesprin-1 is also required for nuclear positioning in muscle syncytial cell formation, where it interacts with the pericentriolar material 1 (PCM-1) protein and microtubule network [52]. Regarding magnetic stimuli, the nucleus seems to have greater susceptibility than the cytoplasm and the exposure to a magnetic field leads to changes in its positioning and cell polarity [53], as well as to morphological changes [54]. Effects on nuclear membrane deformation are also attributed to the increased elasticity, which is higher in the nucleus compared to the plasma membrane thanks to its specific biochemical composition [55]. It was shown that a static magnetic field applied to adipose stem cells (ASCs) leads to changes in nuclear localization during a cell’s differentiation toward the osteogenic lineage, with the nucleus located at the cell’s periphery [42]. Recently, another study used MNPs and magnetic stimulation on MSCs to assess their influence on osteogenic differentiation. The obtained results emphasized the deformation of the nucleus in response to the mechanical tension propagated through focal adhesions, cytoskeleton rearrangements, and ultimately, the nuclear membrane [54].

## 4. Correlation between Nuclear Mechanical Stress, Chromatin Conformational Changes, and Epigenetic Modulation

Epigenetic changes are chemical modifications, usually at the DNA and histone protein level, that ensure the transcriptional regulation of gene expression with the aid of specific proteins. DNA methyltransferases (DNMTs) are enzymes that chemically modify DNA molecules by adding a methyl group (-CH_3_) to cytosine residues, resulting in 5-methylcytosine (5mC); subsequently, oxidization by ten-eleven translocation (TET) enzymes leads to 5-hydroxymethylcytosine (5hmC). DNA methylation is predominantly found at specific sites in the genome, namely, CpG islands, and its occurrence at the gene promotor level is mainly associated with decreased gene expression. Methylation exercises its effects on transcriptional regulation mainly by interfering with the binding of transcription factors. Moreover, a series of successive reactions, starting with TET enzyme activity, can revert DNA methylation [56]. Furthermore, epigenetic regulation at the histone level displays a higher variability and complexity in terms of adding functional groups and the way gene expression is controlled. Histone proteins are susceptible to acetylation, methylation, phosphorylation, and other chemical modifications that modulate their interaction with DNA and, consequently, determine chromatin conformational changes. Therefore, depending on the functional group and its position, histone modification can disrupt these interactions by promoting open chromatin conformation and favoring gene transcription; otherwise, it can enhance them by maintaining heterochromatin and making the DNA inaccessible for RNA polymerases. As a general observation, the acetylation of lysine residues, which is mediated by histone acetylases (HATs), promotes an open chromatin conformation, whereas histone methyltransferases can mediate both the up- and downregulation of gene expression. Similar to DNA methylation, histone modifications are dynamic, reversible epigenetic marks that change their profile depending on cellular needs and their physiological state [57].

Taking into consideration the contact between the LINC components and the nuclear lamins and their role in anchoring chromatin structures, one can assume that morphological changes occurring in cytoskeletons and nuclear membranes might have an influence on chromatin conformation and a cell’s epigenetic profile. In recent years, studies on nuclear mechanosensing have provided evidence to support this idea [58,59,60,61]. Tajik et al. (2016) utilized magnetic beads functionalized with an RGD motif to apply direct mechanical forces at the integrin level and assess the impact on chromatin stretching and transcriptional regulation. Their results highlight the increased transcription in response to chromatin conformational changes mediated by mechanical cues and the essential roles of the LINC components, emerin and lamins, in modulating chromatin mobility [58]. In endothelial cells, chromatin condensation participates in the adaptation to shear stress, with adjustments made simultaneously to the pattern of epigenetic marks at the histone level and to the cytoskeleton rearrangements [58]. The stiffness of the substrate was proven to impact histone deacetylase (HDAC) activity and, consequently, chromatin conformation, as it modulates the transition to myofibroblasts [60] and to osteogenic differentiation [61]. Interestingly, nano-topographical cues seem to participate in the neuronal differentiation of ESCs and MSCs by changing the nuclear morphology and modulating epigenetic marks. A correlation between cytoskeleton rearrangements and nuclear morphology changes could be observed, as well as the differential expression of lamin A/C. For MSCs, such cues further seemed to increase the histone 3 lysine 9 monomethylated (H3K9me1) expression as differentiation progressed, acting in a synergic manner with neuronal inducers. These findings emphasize the crosstalk between the cytoskeleton, nuclear membrane, and epigenetic marks during neuronal differentiation [62]. Furthermore, a relatively limited number of studies investigated the influence of magnetic stimulation and magnetic materials on epigenetic regulation. One recent study assessed the interaction between human submandibular gland cells and maghemite nanoparticles and reported an overall altered epigenetic pattern in DNA methylation and histone 3 (H3) and histone 4 (H4) acetylation that did not induce significant cytotoxic effects. More specifically, the authors reported DNA hypermethylation and the significantly decreased acetylation of H3 and H4 [63]. An electromagnetic field promoted the reprogramming of somatic cells into induced pluripotent stem cells (iPSC) via histone lysine methyltransferase MII2 activity, which increased the levels of histone 3 lysine 4 trimethylation (H3K4me3) that is associated with decondensed chromatin and transcriptional activation [64]. Additionally, electromagnetic field exposure augmented hippocampal neurogenesis in mice, and further in vitro studies on neural stem cells (NSCs) identified increased histone 3 lysine 9 acetylation (H3K9ac) associated with regulatory sequences of *neurod1* and *neurog1* genes [65]. HDAC5 and HDAC11 expression was reported to be modulated, along with multiple other genes during the neuronal differentiation that was induced by the electromagnetic field [66]. Moreover, there are also reports highlighting the possible negative effects of epigenetic modulation in response to magnetic fields. For instance, a 50Hz magnetic field seems to be correlated with changes in the DNA methylation pattern in SH-SY5Y cells and primary cortical neurons, leading to the downregulation of miR-34b and miR-34c. It was observed that the promotor region corresponding to the miR-34b/c primary transcript was hypermethylated after magnetic stimulation. These microRNA molecules participate in regulating redox homeostasis and their decreased expression mediates oxidative damage [67]. Baek et al. (2019) also reported the dysregulation of DNA methylation in vitro during the neuronal differentiation of ESCs. Exposure to a hypomagnetic field interferes with DNMT3B activity, this time causing insufficient methylation and diminishing ESCs’ capacity for differentiation. It was observed that hypomethylated loci corresponded to the promotors of pluripotency factors such as octamer-binding transcription factor 4 (Oct4) and Nanog [68]. Taken together, the scarce number of studies and contradictory results emphasize the need for an extensive analysis on epigenetic regulation, with more focus on the in vivo effects of magnetic field exposure.

## 5. Impact on the Expression of Noncoding RNAs

In recent years, noncoding RNAs have gained much attention as they have proved their essential role in controlling gene expression at the transcriptional and post-transcriptional level, with implications varying from stem cell differentiation to cancer development and progression. Post-transcriptional regulation is usually achieved through short transcripts, such as miRNAs, that mainly act by interfering with mRNAs and blocking their translation [69]. On the other hand, there are also transcripts with regulatory functions that exceed a length of 100 nucleotides and are called long noncoding RNAs (lncRNAs). Thanks to their length, they can form secondary, more complex structures that function as recognition sites for both proteins and nucleic acids. Therefore, they possess a greater versatility and can achieve either transcriptional or post-transcriptional regulation, or can even participate in chromatin remodeling [70]. Studies have shown that magnetic field exposure and MNPs can modify the transcriptional profile of both short and long noncoding RNAs [71,72]. An electromagnetic field of extremely low frequencies can also influence epigenetic control and impact the expression profile of miRNAs in various types of cells, such as pluripotent and adult stem cells, spermatocyte-derived cells, brain cells, and blood cells [73]. Additionally, treatment with MNPs and magnetic stimulation can modulate the paracrine function of MSCs and their exosomal cargo, enriching miRNA content. These exosomes further stimulate wound healing, angiogenesis, and osteogenesis in vivo and in vitro [72,73,74]. For would healing, the effects are mediated via the release of miR-21-5p, which targets sprouty homolog 2 (SPRY2) and activates phosphoinositide 3-kinase (PI3K) and ERK 1/2 signaling in endothelial cells and fibroblasts [72]. On the other hand, exosomes enriched in miR-1260a proved their stimulatory effect on the osteodifferentiation of MSCs and promoted angiogenesis. In endothelial cells, miR-1260a downregulated the expression of the collagen type IV, alpha 2 chain (COL4A2), which is an antiangiogenic factor. More interestingly, osteodifferentiation is promoted by inhibiting HDAC7 expression, which is known to downregulate osteogenic markers such as osteopontin (OPN) and Runt-related transcription factor 2 (RUNX2). Thus, these findings highlight an interplay between the transcriptional and post-transcriptional control of gene expression [74]. The long noncoding RNA INZEB2 is another modulator of osteogenic differentiation which is overexpressed after MSCs interact with MNPs. INZEB2 acts by downregulating the expression of Smad-interacting protein 1 or ZEB2. The authors of the study proposed a mechanism in which INZEB2 prevents ZEB2 from recruiting the C-terminal-binding protein (CtBP) and repressing RUNX2 expression in response to bone morphogenetic protein (BMP) signaling [71]. Additionally, a pulsed electromagnetic field (PEMF) that was applied for 21 days modulated the profile of miRNAs during the osteodifferentiation of MSCs by upregulating miR-26a and miR-29b, and downregulating miR-125b [75].

**Figure 2:**
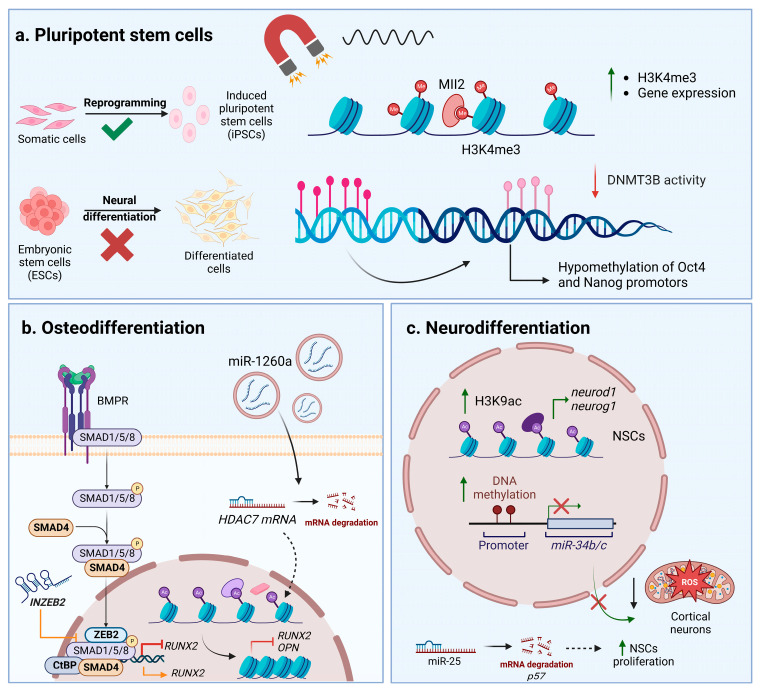
Epigenetic and noncoding RNAs’ control in stem cells exposed to magnetic materials and magnetic fields. (**a**) Influence on pluripotent stem cells. (**b**) Changes during osteodifferentiation. (**c**) Changes during neurodifferentiation. Abbreviations: bone morphogenetic protein receptor—BMPR; C-terminal-binding protein—CtBP; DNA methyltransferase 3B—DNMT3B; embryonic stem cells—ESCs; induced pluripotent stem cells—iPSCs; histone 3 lysine 24 trimethylation—H3K24me3; histone 3 lysine 9 acetylation—H3K9ac; histone deacetylase 7—HDAC7; lysine-specific methyltransferase myeloid/mixed-lineage leukemia 2—Mll2; neural stem cells—NSCs; osteopontin—OPN; reactive oxygen species—ROS; Runt-related transcription factor 2—RUNX2; suppressor of mothers against decapentaplegic—SMAD; Smad-interacting protein 1—ZEB2; long noncoding RNA targeting ZEB2—INZEB. Image was created with BioRender.com.

A scarce number of studies have focused on the influence different types of magnetic stimulation have on noncoding RNAs related to the nervous system. Capelli et al. (2017) exposed peripheral blood cells isolated from patients with Alzheimer’s disease to PEMF and investigated its effects on miRNAs that are known to be involved in this pathology. Even though no significant effects were registered, PEMF could gradually decrease the expression of miR-107, miR-335, and miR-26b as exposure time increased [76]. The downregulation of miR-34b/c in response to a magnetic field was reported in both primary cortical neurons and the SH-SY5Y cell line, whereas expression levels of neuroblastoma-specific miRNAs, such as miR-21-5p, miR-222-3p, and miR-133b, remained unchanged [67,77]. Furthermore, it was observed that repetitive magnetic stimulation (RMS) had beneficial effects toward NSCs, increasing their proliferation and, as a result, upregulating the expression of miRNA molecules such as miR-25 and miR106b, which downregulate p57 and p21 expression [78,79]. These miRNA molecules are part of the miR-106b25 cluster, along with miR-93, that modulate the proliferation and neuronal differentiation of NSCs. The downregulation of miR-25 was associated with a decrease in proliferative capacity, whereas the expression of the whole cluster promoted the expression of the Tuj1 neuronal marker [80]. Interestingly, Cao et al. (2019) observed changes in miRNA-let-7d expression in children with attention deficit hyperactivity disorder (ADHD) after RMS treatment. Serum levels of miRNA-let-7d were significantly increased in ADHD patients compared to healthy children and registered downregulation after magnetic stimulation [81]. Moreover, PEMF can impact the glial differentiation of oligodendrocyte precursor cells in vitro by upregulating miR-219-5p expression and, subsequently, downregulating leucine-rich repeats and immunoglobulin-like domain-containing protein 1 [82]. Figure 2 depicts the crosstalk between epigenetic changes and noncoding RNAs’ regulation of stem cells after interactions between magnetic materials and magnetic fields.

## 6. Important Signaling Pathways Are Activated in Response to Interactions with Magnetic Stimuli and Magnetic Materials

A cellular response to environmental stimuli sensed at a focal adhesion level involves both mechanical changes and the participation of signaling cascades. The Yes-associated protein (YAP)/transcriptional coactivator with PDZ-binding motif (TAZ)-mediated signaling cascades play an important role in mechanotransduction, with implications for the development, organ growth, and lineage specification of MSCs [83,84]. YAP and TAZ are transcriptional regulators that act as effectors for multiple cascades. On stiffer matrices, focal adhesions are more spread out and cells establish more contacts with the substrate, thus creating tension at the cytoskeleton level and creating spatial rearrangements. These events, along with the biochemical modulators, further determine the activation and translocation of the YAP/TAZ factors from the cytoplasm to the nucleus, where they stimulate gene transcription. Otherwise, if cells sense a soft ECM or substrate, mechanical tension is decreased, and YAP/TAZ are relocated to the cytoplasm, where they suffer proteasomal degradation. FAK is a functional component in focal adhesions that is activated when cells sense mechanical stress and forms complexes with members of the Src kinase family. These complexes further trigger a downstream signaling cascade involving multiple phosphorylations, which can regulate actin polymerization and PI3K and ERK pathways, leading to YAP translocation [83,85]. Considering the fact that interactions with magnetic materials and magnetic fields induce mechanotransduction regulation mainly though focal adhesions, YAP/TAZ transcriptional control was reported to be correlated with the biological response of cells [35,54,86,87]. Stem cells cultivated on magnetic scaffolds or exposed to a static magnetic field displayed an increase in YAP/TAZ nuclear localization, which was influenced by the state of actin polymerization and the spatial distribution of the cytoskeleton network [35,86]. YAP/TAZ activation after magnetic stimulation has positive effects on osteogenic differentiation and mineral deposition, but depolymerization of the actin filaments determines cytoplasmic shuttling, highlighting the essential role of the cytoskeleton in cell regulation [86]. Another proposed mechanism during osteogenic differentiation involves the activation of the mitogen-activated protein kinase (MAPK) pathway in response to the mechanical stress induced by MNPs and a magnetic force. Therefore, YAP and RUNX2 transcription factors are translocated to the nucleus, where they upregulate the expression of the collagen type I, alpha 1 chain (COL1A1), OPN, and bone gamma-carboxyglutamate protein (BGLAP) and downregulate the adipogenic marker peroxisome proliferator-activated receptor gamma (PPARG) [54]. Moreover, MAPK-mediated pathways were reported to be activated in response to interactions with magnetic substrates without being correlated with YAP/TAZ transcriptional control. MSCs cultivated on 3D magnetic nanocomposites showed enhanced adherence and an upregulated expression of the integrins FAK and ERK1/2, indicating the activation of this signaling cascade which further promoted the transcription of alkaline phosphatase, OPN, BMP-2, and other osteogenic markers [33]. A static magnetic field was proved to have stimulatory effects toward the proliferation of stem cells, which is mediated by the activation of p38/MAPK and which regulates cytoskeleton reorganization [34]. Furthermore, in mice, an RMS treatment induced the activation of the PI3K/Akt pathway with the subsequent upregulation of glutamate trasporter-1 (GLT-1), allowing for the clearance of glutamate. Thus, a reduction in oxidative stress and neuronal damage was observed, along with improved cognitive function [88]. However, the moderate production of reactive oxygen species (ROS) seems to positively regulate the neuronal differentiation of MSCs induced by electromagnetic field stimulation. The PI3k/Akt pathway is activated in response to EGFR phosphorylation, an event that further determines the phosphorylation of the cyclic AMP response element-binding protein (CREB). The ROS scavenger decreased EGFR activation and inhibited downstream effects, suppressing the neuronal differentiation of MSCs [29].

## 7. The Influence of Magnetic Stimuli and Magnetic Fields on Stem Cell Differentiation and Tissue Engineering Applications

Magnetic cues have been shown to have a great influence on stem cells’ differentiation potential, with increased susceptibility toward the specifications of certain lineages. For example, in the case of ASCs, Maredziak et al. (2016) reported that a static magnetic field increased osteogenic differentiation and the expression of specific markers, but diminished their ability to undergo adipogenesis [41]. Indeed, many studies have approached magnetic stimulation by concentrating on osteogenic differentiation. The beneficial effects observed in this type of specification might be attributed to the mechanical characteristics of the bone, which is a hard tissue. Rigid matrices with reduced elasticity were proven to influence and direct the differentiation of MSCs toward an osteogenic lineage [20]. Looking into MSCs, the mechanosensing machinery of the cells can dictate their differentiation toward osteoblasts or adipocytes by modulating YAP/TAZ transcriptional regulation depending on the stiffness of the substrate [83]. Furthermore, there is evidence indicating that osteogenesis is positively regulated by the overexpression of integrins, which is accompanied by changes in cytoskeleton arrangement and the activation of specific pathways [89].

**Figure 3:**
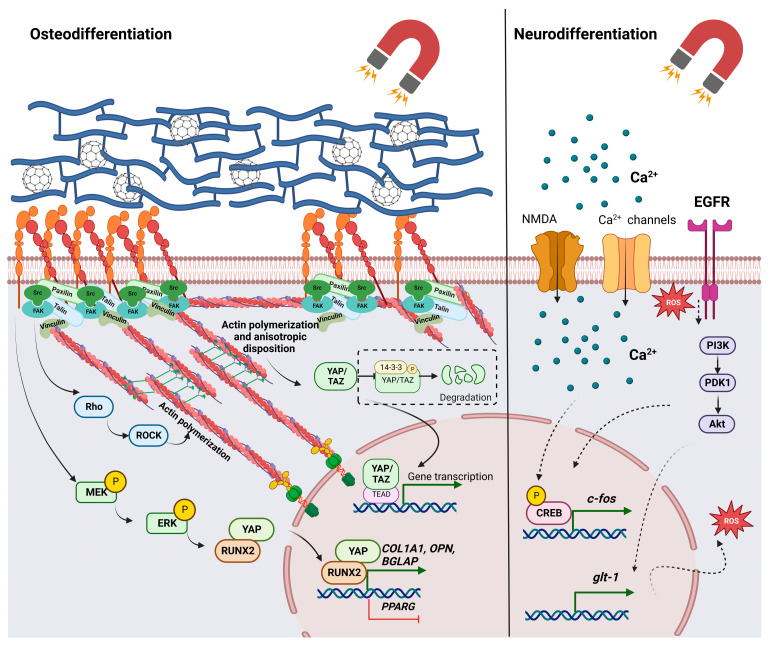
Signaling pathways associated with stem cell differentiation in response to magnetic materials and magnetic fields. Abbreviations: protein kinase B—Akt; bone gamma-carboxyglutamate protein—BGLAP; calcium ions—Ca^2+^; cAMP response element-binding protein—CREB; collagen type I, alpha 1 chain—COL1A1; extracellular signal-regulated kinases—ERKs; focal adhesion kinase—FAK; glutamate transporter-1—GLT-1; MAP kinase kinase—MEK; N-methyl-D-aspartate calcium channel—NMDA; osteopontin—OPN; phosphoinositide-dependent kinase-1—PDK1; phosphoinositide 3-kinase—PI3K; peroxisome proliferator-activated receptor gamma—PPARG; Ras homologous—Rho; Rho-associated protein kinase—ROCK; reactive oxygen species—ROS; transcriptional coactivator with PDZ-binding motif—TAZ; TEA domain transcription factor 1—TEAD1; Yes-associated protein—YAP). Image was created with BioRender.com.

Considering the previously discussed effects of magnetic stimulation on mechanotransduction, magnetic cues and materials have emerged as a valuable resource for the promotion of osteogenesis, with great potential in bone tissue engineering [33,90]. For example, MNPs were incorporated into a calcium phosphate cement by obtaining an osteoinductive scaffold that was seeded with human dental pulp stem cells [91]. Exposure of this bioconstruct to a static magnetic field significantly increased the osteogenic differentiation of stem cells, as shown by the increased expression of osteogenic markers and alkaline phosphatase activity. Further, differentiated cells had higher yields of bone mineral synthesis compared to those seeded on calcium phosphate cement alone or on unmagnetized scaffolds [91]. Furthermore, in vivo studies showed promising results for both magnetic materials and magnetic fields in promoting bone regeneration. Gene therapy was used to stimulate angiogenesis and osteogenesis after a bone implant in rabbits. Magnetic microspheres and magnetic fields were used to facilitate the transfection of plasmids expressing vascular endothelial factor (VEGF) [92]. In diabetic mice, a static magnetic field prevented trabecular and cortical bone deterioration, increased the number of osteoblasts, and decreased the proportion of osteoclasts. Additionally, the magnetic field treatment led to the enhanced expression of bone-specific proteins, such as BMP and osteocalcin [90]. Possible applications for musculoskeletal regeneration were reported regarding chondrogenic [87,93], tenogenic [35], and myogenic [94] differentiation. It seems that magneto-responsive scaffolds and magnetic fields have synergistic effects that promote tenogenesis by increasing specific markers such as tenomodulin, scleraxis, and decorin [35,95]. This approach, combined with the activation of activin receptor type II (ActRIIA), allowed for the modulation of the transforming growth factor β (TGF-β)/Smad2/3 signaling pathway [95]. Other experiments were also conducted for the purpose of studying cardiac [96], vascular [97], and nervous tissue [11] approaches. Figure 3 shows the important signaling pathways that were reported during osteodifferentiation and neurodifferentiation.

## 8. Applications of Magnetic Nanoparticles, Magnetic Materials, and Magnetic Fields for Nervous Tissue Regeneration

### 8.1. Impact on Cell Behavior and Neuronal Differentiation

Studies on neurogenesis emphasize its promising results for the use of magnetic fields for increasing NSC differentiation, proliferation, and maturation [65,98]. These effects might be mediated by calcium channels’ activity and calcium signaling, which is suggested by the upregulated expression of the Ca(v)1 channel and increased cyclic AMP response element-binding protein (CREB) phosphorylation. The inhibition of the Ca(v)1 channel significantly decreased the differentiation and maturation of NSCs [99]. Another calcium channel, N-methyl-D-aspartate (NMDA), was reported to be upregulated during the differentiation of neural progenitor cells, leading to the overexpression of the CREB-regulated c-fos protein [100]. Furthermore, in embryonic NSCs, electromagnetic field exposure promotes neurodifferentiation through the upregulation of transient receptor potential canonical 1 (TRPC1), which modulates Ca^2+^ uptake. The increase in intracellular calcium levels was accompanied by the upregulation of NeuroD and Neurogenin1 [101]. An electromagnetic field was reported to promote the neuronal differentiation of MSCs and NSCs, and was accompanied by phenotypic and electrophysiological changes. Transcriptome analysis of both cell types highlighted significant changes in the global gene expression profile that were mediated by the upregulation of transcription factors such as hairy and enhancer of split-1 (HES1), early growth response protein-1 (Egr1), and DNA-binding protein inhibitor ID1. Among them, Egr1 proved to be a key regulator that synergizes with the electromagnetic field exposure to promote neuronal differentiation [66]. MNPs internalization with further magnetic stimulation promoted in vitro neural differentiation of ESCs, synergizing with biochemical inducers [102], and allowed guidance of neurite outgrowth for primary leech neurons [103]. Besides neuronal differentiation, some studies indicated magnetic stimulation and MNPs as a potential approach for nerve TE and spinal cord injuries [104,105,106]. Such cues were shown to promote peripheral nerve regeneration in rats by increasing the survival of dorsal root ganglia (DRG) neurons, and improving myelination and functional recovery [104,105]. These outcomes are also mediated via stimulation of a pro-regenerative phenotype of Schwann cells, which have a critical role during repair since they secrete neurotrophic factors and are responsible for myelination [107,108].

### 8.2. Cell Labeling and Guidance

Interactions established between cells and MNPs have created the opportunity for directed cell delivery for therapeutic purposes. This aspect is of high interest for nervous TE since it could facilitate cell transplantation at inaccessible lesioned sites. For this purpose, MSCs were investigated as promising candidates for spinal cord injuries. Labeling MSCs with superparamagnetic iron oxide nanoparticles (SPIONs) proved to have no significant cytotoxic effects and allowed for in vivo tracking using magnetic resonance imaging (MRI) [109]. Cell guiding was achieved by Tukmachev et al. (2015) who developed a noninvasive magnetic system with two magnets flanking the spinal cord over the injury site. MSCs were labeled with SPIONs coated with poly-L-lactic acid and intrathecally administered near the lesioned area. Results from a histological analysis showed that the presence of magnetic stimulations led to MSC concentration and infiltration at the lesioned site, whereas in its absence, cells were evenly distributed throughout the spinal cord [110]. For brain injuries, NSCs were labeled and used for cell therapy. Studies showed that nanocomposites containing SPIONs do not affect NSCs’ potential of differentiation toward both neurogenic and glial lineages [111], whereas magnetically guided NSCs display better viability in vivo and have a precise location at the brain level [112].

### 8.3. Gene and Drug Delivery

Given the low capacity of nervous tissue to regenerate, bioactive molecules emerged as efficient therapeutics to stimulate the regeneration process. Therefore, strategies for precise administration of such molecules are being investigated for the central and peripheral nervous system, but they represent a real challenge. When designing magnetic vehicles for gene and drug delivery, one must take into consideration if they target the peripheral nervous system (PNS) or central nervous system (CNS), since passing the brain–blood barrier (BBB) or blood–cerebral spinal fluid barrier requires different modifications than for PNS administration. In cases of damaged tissue, MNPs could pass CNS barriers freely, but for neurodegenerative disorders, they might need functional groups to bind cell receptors [113]. MNPs functionalized with osmotin could pass the BBB without inducing any damage or cytotoxic effects. Moreover, they could attenuate memory and tau phosphorylation in an Alzheimer model [114]. Furthermore, Niu et al. (2017) combined small RNA with growth factor delivery and obtained promising results in a Parkinson model. MNPs were functionalized with nerve growth factor (NGF) and short hairpin RNA (shRNA) against α-synuclein, decreasing the number of α-synuclein-positive neurons [115].

### 8.4. Scaffold-Based Approaches

Scaffold-based approaches for nervous tissue regeneration were mainly utilized for the peripheral system and spinal cord injuries, as these compartments require precise cell alignment and are protected by connective tissues. MNPs are usually incorporated into polymeric scaffolds, with hydrogels being intensely used [1,113]. The concentration of MNPs is an important factor that needs optimization, as it can affect cell viability and differentiation. Collagen-based coatings enriched with 0.5% MNPs showed better results in terms of cell adherence, viability, and the neural differentiation of pluripotent stem cells as compared to higher contents of up to 4% [116]. Furthermore, xanthan-based scaffolds with moderate contents of MNPs promoted cell adhesion and proliferation. Interestingly, while neat scaffolds presented a higher percentage of differentiated neurons expressing microtubule-associated protein 2 (MAP2) and Tuj1, materials with MNP content displayed more cells that were positive for synaptophysin and increased electrical transmission. Therefore, it can be assumed that the presence of MNPs stimulates the differentiation of functional neurons [117]. Rose et al. (2017) developed a scaffold-based system, called Anisogel, based on SPIONs. Microgels were loaded with SPIONs and dispersed into a hydrogel matrix precursor, and then were aligned by applying an external magnetic field. The obtained system was further tested on fibroblasts and nerve cells. This approach allowed for the precise control over the topographical and mechanical cues necessary to direct cell growth. Compared to randomly oriented microgels, cells cultivated on the aligned microgel system displayed a better orientation of neurite outgrowth and they grew in an aligned way, emphasizing the good effects of the pre-existing topographical cues. [11]. SPION-loaded polycaprolactone (PCL) nanofibers, which were magnetically oriented to form an injectable hydrogel, could support and enhance the neural differentiation of olfactory ectomesenchymal stem cells. The anisotropic nature of the resulting hydrogels, which shares similarities with the nerve tissue architecture, in addition to the presence of magnetic structures, might mediate the beneficial effects on stem cell differentiation [8]. Consistent with these results, nanotopographical cues were reported to play an important part during the neuronal differentiation of ESCs and MSCs, with an influence on nuclear morphology and epigenetic regulation in the first 24 h of a cell–substrate interaction [62]. Another magnetic hydrogel system was developed by Tay et al. (2018), allowing for the neuromodulation of primary dorsal root ganglion neurons. It was observed that a short magnetic stimulation led to the activation of transient receptor potential vanilloid (TRPV) and piezo-type mechanosensitive ion channel component 2 (PIEZO2) channels, increasing calcium uptake [12].

## 9. Limitations Concerning the Use of Magnetic Materials and Magnetic Stimulation

Magnetic materials and magnetic stimulation represent a relatively new approach in biomedical applications and TE. Even though numerous beneficial effects have been reported, there are also studies that have found a negative impact and raise questions regarding their limitations and optimal conditions of use [73]. For MNPs, one particular topic of interest is represented by concentration, since nanoparticle internalization was associated with alterations in redox homeostasis, an increase in ROS production, and diminished viability [103,118,119]. It was observed that cytotoxic effects also depend on cellular type and the type of MNPs used [103,120]. Higher concentrations of MNPs, along with magnetic field exposure, can cause disruptions in focal adhesions and the cytoskeleton network [26,27]. Moreover, unoptimized conditions can interfere with neural differentiation [116]. With respect to magnetic stimulation, concerns are being raised regarding intensity, frequency, and time of exposure [74,116,121]. Indeed, several studies reported that prolonged exposure to magnetic stimulation can affect cell viability [102] and molecular regulation [121]. Erdal et al. (2018) assessed the influence of an electromagnetic field (50 Hz, 1 mT) on miRNA expression in rats. The obtained results showed that a long exposure of up to 60 days differentially altered the expression profile of miRNAs that are involved in certain neurological disorders (miR-9-5p, miR-26b-5p, miR-29a-3p, miR-106b-5p, miR-107, and miR-125a-3p) depending on sex, age, and cell type [121]. Studies on the SH-SY5Y cell line showed contradictory results regarding the beneficial effects of magnetic fields and their use for neurodegenerative disorders, including for sensitizing cells to a Parkinson’s inducer [122]. These findings further emphasize the need for a better understanding of the effects associated with magnetic stimulation and the optimization of the conditions of exposure such as time, frequency, or intensity.

## 10. Conclusions

Studies conducted thus far indicate the role of magnetic materials and magnetic stimulation in modulating mechanotransduction pathways that, in turn, seem to have a more profound influence on molecular mechanisms. Focal adhesion complexes and the cytoskeleton network are highly impacted by these environmental cues and the essential players that elaborate specific responses. Structural changes occurring in these compartments participate in the mechanical deformation of the nucleus and were correlated with the modulation of signaling pathways and gene expression. Additionally, mechanical changes at the cellular level were shown to regulate epigenetic mechanisms, and recent studies have indicated the influence of magnetic stimulation on DNA methylation, histone modifications, and the profile of noncoding RNAs in stem cell differentiation. Taken together, these findings pave the way for deciphering how intracellular systems interact with each other in response to magnetic substrates and magnetic stimulation. Moreover, in nervous tissue engineering, these factors are valuable tools since they can be used to develop complex systems by mimicking the tissue microenvironment or they can ease therapeutic access. However, such approaches are relatively new and there is a need to understand to what extent these therapies can have a beneficial impact, as well as a need to optimize the existing methods.

## Data Availability

Not applicable.
